# O6.2. NEUROBIOLOGY OF PSYCHOMETRIC SCHIZOTYPY: INSIGHTS FROM MULTIMODAL IMAGING RESEARCH

**DOI:** 10.1093/schbul/sby015.223

**Published:** 2018-04-01

**Authors:** Gemma Modinos, Alice Egerton, David J Lythgoe, Gareth J Barker, Katrina McMullen, Christian Keysers, André Aleman, Veena Kumari, Steven CR Williams

**Affiliations:** 1 Institute of Psychiatry, Psychology & Neuroscience, King’s College London; 2 Netherlands Institute for Neuroscience, Netherlands Academy for Arts and Sciences, KNAW, University of Amsterdam; 3 University of Groningen, University Medical Hospital Groningen

## Abstract

**Background:**

The continuum approach to psychosis proposes a dimensional continuity between the neurobiology of subclinical psychotic-like experiences in healthy individuals (or schizotypy) and psychotic symptoms in clinically relevant psychosis (Linscott and van Os, 2013, Nelson et al., 2013). Preclinical models propose that cortical glutamate dysfunction related to cortico-limbic-striatal hyper-responsivity to stress may underlie both hippocampal and striatal overdrive as well as gray matter loss associated with schizophrenia-like behaviors (Berretta et al., 2001, Lodge and Grace, 2011). Our recent studies investigated whether changes in brain glutamate are present in healthy individuals with high psychometric schizotypy, and whether these are related to changes in (1) corticolimbic response to emotion and (2) gray matter volume (GMV).

**Methods:**

Forty-eight healthy participants were recruited based on their score on the O-LIFE questionnaire (Mason et al., 2005), after pre-screening 250 respondents to online advertisement. Participants with high levels of unusual experiences (HS group; that is, scored >7 on the Unusual Experiences (UE) subscale of the O-LIFE), and participants with low UE (LS group; that is, <2 on O-LIFE UE subscale), were invited to participate. Groups were matched by age, gender and IQ. A structural MRI scan, glutamate proton magnetic resonance spectroscopy in the anterior cingulate cortex (ACC), and functional magnetic resonance imaging (fMRI) measuring corticolimbic response during emotional processing were acquired at 3T in a single session. Glutamate levels were analyzed using LCModel 6.3-1L. Voxel-based morphometry was applied to quantify GMV and both GMV and fMRI group level analyses were run using SPM12. Standalone imaging results as well as fMRI/sMRI × glutamate interactions were considered significant after voxel-wise P<0.05 family-wise error correction.

**Results:**

While viewing emotional pictures, HS individuals showed greater activation than did subjects with LS in the caudate, and marginally in the ACC, hippocampus, medial prefrontal cortex (MPFC) and putamen. Although no between-group differences were found in glutamate concentrations, within the HS group ACC glutamate was negatively correlated with striatal activation (bilaterally in caudate and in left putamen at P < 0.05) and marginally with MPFC (P = 0.052) and amygdala (left: P = 0.062; right: P = 0.079), correlations that were not present in LS subjects. Structurally, subjects with HS showed GMV decreases in the rolandic operculum/superior temporal gyrus (P < 0.05) at the whole-brain level, and significant increases in GMV were also detected using ROI in the precuneus and ACC (both P < 0.05). Furthermore, in HS subjects ACC glutamate levels were negatively correlated with GMV in the ACC (P < 0.05). Such association was absent in LS. These findings provide, to our knowledge, the first evidence that brain glutamate levels are associated with emotional hyper-responsivity and volumetric changes in HS in brain regions thought to be critical in the pathophysiology of psychotic symptoms.

**Discussion:**

Collectively, these results are in line with a dimensional view of psychosis by suggesting that interactions between brain structure, neurochemistry, and functional response to emotion within a corticolimbic circuit are involved in the expression of psychotic-like experiences at non-clinical and clinical levels. These findings may also serve as evidence of potentially protective mechanisms, as our studies involved high-functioning individuals with HS and some of the observed effects are opposite to what would have been predicted from studies in clinical groups.

